# Laparoscopic Fenestration for a Hepatic Cyst Guided by Preoperative 3D Simulation: A Challenging Case for Fenestration Site Selection

**DOI:** 10.1111/ases.70294

**Published:** 2026-04-20

**Authors:** Fumiya Hasegawa, Daisuke Noguchi, Takahiro Ito, Aoi Hayasaki, Yusuke Iizawa, Takehiro Fujii, Akihiro Tanemura, Yasuhiro Murata, Naohisa Kuriyama, Masashi Kishiwada, Shugo Mizuno

**Affiliations:** ^1^ Department of Hepatobiliary Pancreatic and Transplant Surgery Mie University Graduate School of Medicine Tsu Japan

**Keywords:** 3D simulation, hepatic cyst, indocyanine green, laparoscopic fenestration

## Abstract

Fenestration is the standard treatment for hepatic cysts, but recurrence remains a concern. We report the case of a 73‐year‐old woman who presented with leg edema. Computed tomography revealed a 140‐mm hepatic cyst extending from segments 8 to 1, compressing the inferior vena cava (IVC). Preoperative 3D simulation using artificial intelligence‐assisted imaging software (SYNAPSE VINCENT) demonstrated that the exposed area of the cyst wall not covered by liver parenchyma was limited to the space part of the caudate lobe behind the hepatoduodenal ligament. Laparoscopic fenestration was performed with intraoperative indocyanine green (ICG) guidance to avoid injury to the liver and biliary tract while maximizing the fenestration area. The patient experienced no postoperative complications, and no symptomatic recurrence was observed during 6 months of follow‐up. This case highlights the utility of preoperative 3D simulation for fenestration site selection in a challenging hepatic cyst.

## 1 Introduction

Hepatic cysts are generally treated when symptomatic, with laparoscopic fenestration being a minimally invasive and effective standard procedure. The postoperative recurrence rate has been reported to be 9.6% [1], and factors such as the number and location of cysts are thought to be associated with recurrence [2]. Therefore, appropriate preoperative assessment and individualized surgical planning are crucial.

## 2 Case Presentation

**FIGURE 1:**
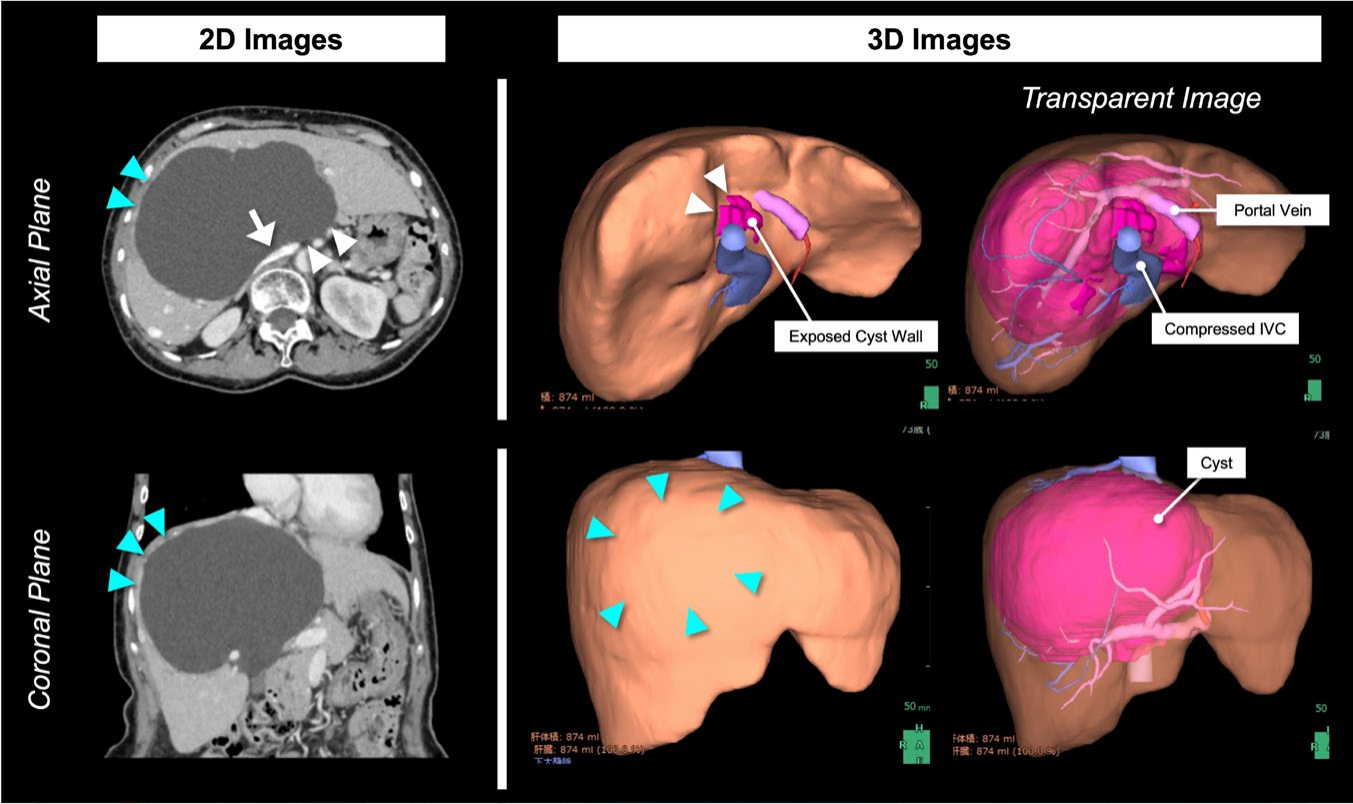
Preoperative imaging: 2D and 3D. A 140‐mm cyst was primarily located in segment 8 of the liver, extending exophytically and partially into the caudate lobe, and compressing the IVC (arrow). Two potential fenestration sites were suggested on 2D images: the ventral surface of the cyst (blue arrowhead) and a limited area of the caudate lobe behind the hepatoduodenal ligament (white arrowhead). 3D reconstruction using AI‐based software (SYNAPSE VINCENT) highlighted the exposed cyst wall (pink) and showed that the ventral surface was covered by liver parenchyma (blue arrowhead). Based on the intuitive understanding of the cyst's location, size, and extent from the 3D images, the target cyst wall for fenestration (white arrowhead) was selected preoperatively. The relationship between the cyst and the intrahepatic vascular structures was visualized using a transparency‐adjusted liver image. 2D, two‐dimensional; 3D, three‐dimensional; AI, artificial intelligence; IVC, inferior vena cava.

**FIGURE 2:**
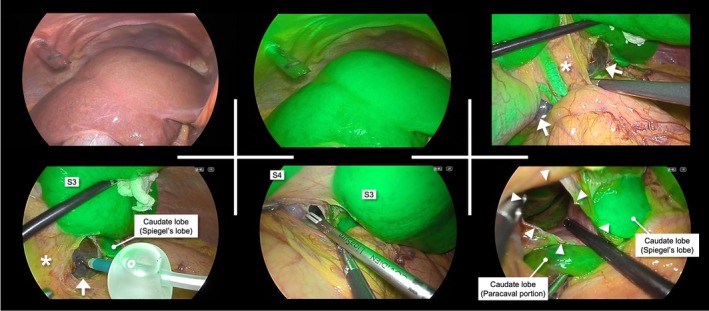
Intraoperative findings. The anterior surface of the cyst was covered by liver parenchyma as confirmed by intraoperative ultrasonography and ICG, consistent with the preoperative simulation. After incising the lesser omentum and entering the omental sac, the cyst wall (arrow) was exposed behind the hepatoduodenal ligament (*). Following cyst puncture and aspiration, dissection was performed along the boundary between the cyst wall and the green‐fluorescent liver parenchyma under ICG fluorescence guidance, enabling maximal fenestration (arrowhead) while avoiding injury to the liver parenchyma and bile ducts. ICG, indocyanine green.

**FIGURE 3:**
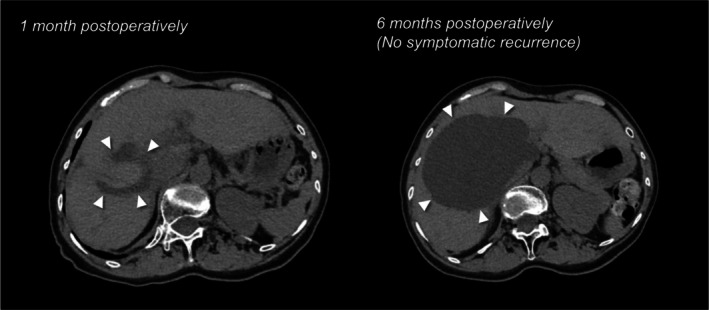
Postoperative CT findings in the present case. Postoperative CT at 1 month (left image) demonstrated marked reduction of the hepatic cyst (arrowheads). At 6 months postoperatively (right image), radiologic enlargement was observed (arrowheads), while the cyst remained smaller than its preoperative size and no recurrence with symptoms was noted.

A 73‐year‐old woman presented with a 1‐year history of progressive bilateral leg edema. Physical examination showed a soft abdomen without tenderness and pitting edema of both legs. Laboratory tests showed elevated hepatobiliary enzymes: AST 49 IU/L, ALT 59 IU/L, and ALP 135 IU/L. Contrast‐enhanced computed tomography (CT) revealed a 140‐mm cyst primarily located in segment 8 to 1 of the liver, extending exophytically and partially reaching the caudate lobe. The cyst compressed the inferior vena cava (IVC), which was considered the cause of the leg edema. Mild intrahepatic bile duct dilatation was also observed. Magnetic resonance imaging (MRI) did not show solid components, and communication with the bile ducts was not evident. Laparoscopic fenestration was planned for a symptomatic hepatic cyst. To determine the optimal site and extent of fenestration preoperatively, we generated three‐dimensional (3D) reconstructed images of the liver from CT data using artificial intelligence (AI)‐based image analysis software (SYNAPSE VINCENT, Fujifilm) and evaluated the cyst wall not covered by liver parenchyma in three dimensions. Conventional two‐dimensional (2D) CT suggested two potential fenestration sites: one on the ventral surface of the anterior segment and another at the caudate lobe, located between the hepatoduodenal ligament and the IVC (Figure 1). However, 3D reconstruction provided an intuitive understanding of the cyst's position and extent, allowing evaluation of fenestration sites from all directions. This revealed that the ventral anterior segment remained covered by thin hepatic parenchyma, leaving only the area in the caudate lobe truly exposed. Based on this simulation, we planned the cyst wall in the caudate lobe as the target for fenestration, and, if an adequate window could not be achieved, to perform additional parenchymal transection on the anterior surface for an effective drainage route. To maximize the window while avoiding liver and biliary injury, ICG fluorescence guidance was employed; 2.5 mg of ICG was intravenously administered at the induction of anesthesia. Intraoperative findings are shown in Figure 2. The anterior surface of the cyst was covered by up to 10 mm of hepatic parenchyma, as confirmed by intraoperative ultrasonography and ICG. After opening the lesser omentum, as anticipated preoperatively, the exposed cyst wall was identified behind the hepatoduodenal ligament. The cyst was punctured and aspirated, yielding approximately 900 mL of fluid. Under ICG fluorescence, the boundary between hepatic parenchyma and the cystic wall was clearly delineated, enabling safe maximal fenestration (70 × 20 mm) using laparoscopic coagulating shears. No bile leakage was observed at the fenestration site, which was also confirmed using ICG fluorescence imaging. An anti‐adhesion material was applied to the fenestrated cavity. The operation time was 99 min with minimal blood loss. Postoperatively, the patient's leg edema resolved on postoperative day (POD) 1, and no bile leakage was observed. On POD 4, hepatobiliary enzymes were normalized (AST 25 IU/L, ALT 26 IU/L, ALP 85 IU/L), and the patient was discharged. No symptomatic recurrence was noted during the 6‐month follow‐up period, with radiological re‐enlargement observed (Figure 3).

## 3 Discussion

Our experience suggests that preoperative 3D simulation facilitates a tailored surgical strategy in challenging hepatic cyst cases and helps secure an appropriate fenestration site safely.

**FIGURE 4:**
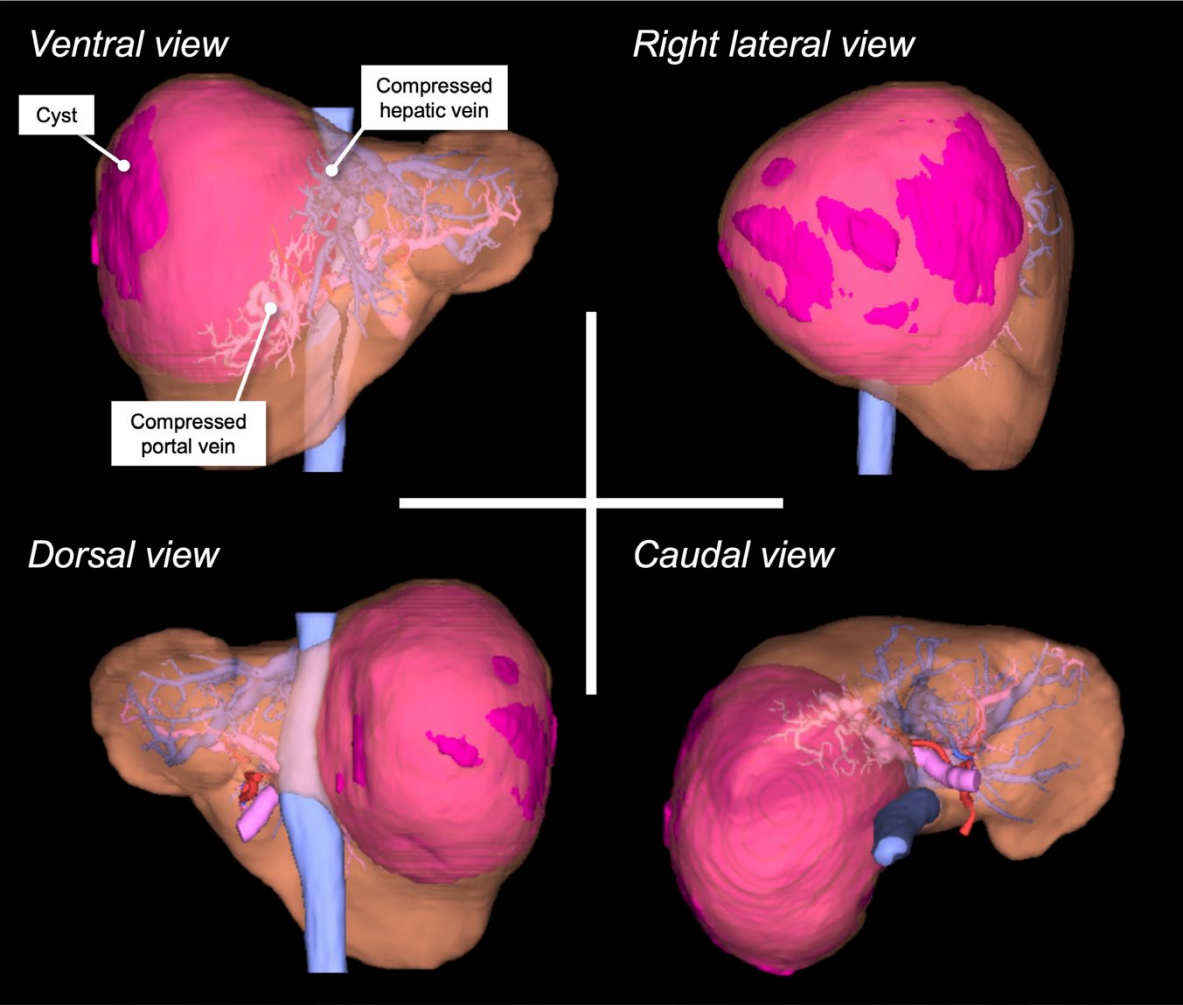
3D simulation of Case 3. A cyst extending from segment 7 to 6 (maximum diameter, 162 mm; volume, 1430 mL) was shown, causing dorsal compression of the portal vein and hepatic vein. Based on the 3D simulation, right dorsal liver mobilization was anticipated, and the patient's position was preoperatively set to the left semi‐lateral position. Images were shown from the ventral, right lateral, dorsal, and caudal perspectives.

Although laparoscopic fenestration is currently the standard minimally invasive approach, inherent blind spots remain due to the restricted laparoscopic field of view, particularly in cases with large cysts. Preoperative 3D simulation complements these limitations by providing a more accurate and intuitive understanding of cyst anatomy than conventional 2D imaging, closely reflecting intraoperative findings [3]. Between 2008 and 2024, 44 patients underwent hepatic cyst surgery at our institution, including 38 fenestrations (35 laparoscopic and 3 open) and 6 hepatectomies. After the introduction of AI‐based imaging software in 2017, preoperative 3D simulation was selectively performed at the surgeon's discretion in technically challenging cases (e.g., deep/caudate, large or multiple, perivascular/peribiliary, or recurrent lesions) when considered necessary and was applied in 7 of 35 laparoscopic fenestrations (Table S1). In our series, preoperative 3D simulation was useful for (i) complementing the limitations of laparoscopy and (ii) determining patient positioning and port placement. As an example of (ii), in a case requiring right dorsal liver mobilization (Case 3 in Table S1), preoperative planning with the left semi‐lateral position facilitated team‐based strategy sharing and safe operation (Figure 4). In the present case, despite limited cyst wall exposure, detailed 3D evaluation additionally enabled identification of the optimal fenestration site in the caudate lobe. To our knowledge, this is the first case in our series, as well as in the literature, in which the fenestration site itself was preoperatively identified. As in liver resection, the technical difficulty of fenestration depends largely on cyst location and extent, necessitating individualized surgical planning. Based on this experience, preoperative 3D simulation could help the surgical planning of hepatic cyst cases.

Among the 38 fenestration cases, symptomatic recurrence occurred in three cases (8%), whereas no recurrence was observed in the subset of seven laparoscopic fenestration cases with preoperative 3D simulation (Table S1). Recurrence was defined based on symptoms, a clinically meaningful endpoint consistent with previous reports [4, 5]. Patients were followed for up to 6 months; some were referred earlier to their physicians with instructions to return if symptoms recurred. Radiological re‐enlargement was seen in the present case. Following preoperative 3D simulation, refinement of the fenestration technique may be the next step.

While wider fenestration is considered effective in preventing postoperative recurrence [6], a more extensive opening may increase the risk of liver parenchymal and bile duct injury [7]. ICG fluorescence imaging allows real‐time visualization of the liver parenchyma and biliary anatomy, helping prevent these injuries while safely maximizing the fenestration area, and more reports have supported its utility in hepatic cyst surgery in recent years [8, 9]. In our series, this technique was applied in 4 of 38 fenestration cases; none developed bile leakage or symptomatic recurrence, including the present case. In the present case, ICG was administered intravenously at induction of general anesthesia (approximately 60 min before observation). The optimal timing of ICG administration has not yet been established and remains an important subject for future investigation [10]. When biliary communication or intraoperative bile leakage is identified, we consider modifying the surgical strategy, including conversion to hepatectomy if necessary. To date, all intraoperative bile leaks at our institution have been successfully managed with primary closure.

This report underscores the value of 3D preoperative simulation in selecting the optimal fenestration site in challenging hepatic cysts and may support future cyst surgery.

## Author Contributions

**F.H**., **D.N**., and **S.M.:** conception and design of the work. **F.H**., **D.N**., **T.I**., **A.H**., **Y.I**., **T.F**., **A.T**., and **Y.M.:** acquisition and analysis of data. **F.H**. and **D.N.:** drafting of the manuscript. Critical revision of the manuscript for important intellectual content: All authors, with major contributions from K.N., M.K., and S.M. All authors have read and approved the final version of the manuscript and agree to be accountable for all aspects of the work in ensuring that questions related to the accuracy or integrity of any part of the work are appropriately investigated and resolved. The authors declare no proprietary or commercial interest in any product mentioned or concept discussed in this article.

## Ethics Statement

This study was approved by the institutional ethics committee of Mie University Hospital (approval number: H2025‐131) and conducted in accordance with the latest revision of the Declaration of Helsinki.

## Conflicts of Interest

The authors declare no conflicts of interest.

## Supporting information


**Table S1:** Laparoscopic fenestration cases undergoing preoperative 3D simulation.

## Data Availability

The data that support the findings of this study are available from the corresponding author upon reasonable request.
