# Supplementary material for: Laparoscopic Fenestration for a Hepatic Cyst Guided by Preoperative 3D Simulation: A Challenging Case for Fenestration Site Selection
Source: Asian J Endosc Surg. 2026 Apr 20;19:e70294. doi: 10.1111/ases.70294 (PMC13096759; doi:10.1111/ases.70294)
Supplement: Supplementary file 1 — Table S1: Laparoscopic fenestration cases undergoing preoperative 3D simulation. [file ASES-19-e70294-s001.docx]

**Supplemental Table. Laparoscopic fenestration cases undergoing preoperative 3D simulation**

| **#** | **Year** | **Age/Sex** | **Symptoms** | **Location of target cyst^a^** | **Maximum diameter of target cyst (mm)** | **Fenestrated site^b^** | **Patient position** | **Operation time (min)** | **Complication** | **Symptomatic recurrence** | **Follow-up period**  **(month)** |
| --- | --- | --- | --- | --- | --- | --- | --- | --- | --- | --- | --- |
| 1 | 2017 | 68/M | RUQ pain | S5–S6 | 138 | S5 | Lithotomy | 225 | None | No | 68 |
| 2 | 2018 | 54/F | RUQ pain | S7–S8–S5–S6 | 177 | S7 | Left semi-lateral | 237 | None | No | 20 |
| 3 | 2019 | 45/M | Right flank pain | S7–S6 | 162 | S7 | Left semi-lateral | 318 | None | No | 2 (Follow-up at another hospital) |
| 4 | 2022 | 77/M | Abdominal distension | S5–S6 | 95 | S5 | Split-leg | 85 | None | No | 15 |
| 5 | 2023 | 60/F | Abdominal pain | #1: S6  #2: S7 | #1: 125  #2: 115 | #1: S6  #2: S7 | Left semi-lateral | 193 | None | No | 0 (Follow-up at another hospital) |
| 6 | 2024 | 58/F | Epigastric pain | S3 | 80 | S3 | Split-leg | 98 | None | No | 1 (Follow-up at another hospital) |
| 7  (Present case) | 2024 | 73/F | Leg edema | S8–S1 | 140 | S1 | Split-leg | 99 | None | No | 6 |

a. Liver segments were defined according to Couinaud’s classification. The target cyst was defined as the symptomatic cyst requiring treatment. Based on preoperative CT findings, the segment in which the target cyst was primarily located is listed first.

b. Fenestrated site is described based on intraoperative findings.

Abbreviations: RUQ pain, right upper quadrant pain.
